# Bilateral Shoulder Infections and Delayed Recognition of Spinal Discitis in an Adult Male With Mild COVID-19 and Staphylococcus aureus Bacteremia: Case Report and Literature Review

**DOI:** 10.7759/cureus.74765

**Published:** 2024-11-29

**Authors:** John G Skedros, Jessie A Montgomery, John T Cronin, Brett W Richards, Kevin B Curtis, Michelle M Matheu, Mark Mulcaire-Jones

**Affiliations:** 1 Shoulder and Elbow, Utah Orthopaedic Specialists, Salt Lake City, USA; 2 Medical Education, University of Utah School of Medicine, Salt Lake City, USA; 3 Infectious Disease, Intermountain Medical Center, Salt Lake City, USA; 4 Internal Medicine, University of Michigan, Ann Arbor, USA

**Keywords:** bacterial co-infection, bilateral shoulder infection, covid-19, methicillin-sensitive staphylococcus aureus, vertebral discitis

## Abstract

Coronavirus disease 2019 (COVID-19) has been associated with numerous complications beyond the respiratory tract, including spinal and joint co-infections and secondary infections. However, we could not locate any reported cases of bilateral shoulder infections with additional spinal infections associated with mild (like our patient) COVID-19 or with more severe cases of COVID-19. We report the case of a healthy 62-year-old male who presented with mild COVID-19 and concurrent methicillin-sensitive *Staphylococcus aureus *(MSSA) bacteremia associated with multiple musculoskeletal sites of deep-seated infection. These included bilateral shoulder infections and delayed detection of lumbar spondylodiscitis with spinal epidural abscess (SEA). The patient had no known risk factors for complicated MSSA bacteremia. We discuss the potential pathophysiology and highlight the increased risk for worse outcomes in patients with mild COVID-19 and bacterial co-infections. This case is not only unique but provides valuable insight for physicians tasked with making difficult diagnoses that may be masked or complicated by COVID-19 infection.

## Introduction

Background 

Coronavirus disease 2019 (COVID-19) is a clinical syndrome resulting from infection with severe acute respiratory syndrome coronavirus 2 (SARS-CoV-2). While COVID-19 primarily affects the respiratory tract, its effects have been demonstrated in most organ systems, including the heart, kidneys, liver, central nervous system, and others [1,2]. Co-infections and secondary infections with other pathogens are common for patients infected with SARS-CoV-2. Most of the cases described in the literature are in patients requiring hospitalization for COVID-19. There are numerous case studies reporting concurrent musculoskeletal bacterial infections in patients with COVID-19. These include spinal disc and bone infections (spondylodiscitis/osteomyelitis), spinal epidural abscess (SEA), and infections of the sternoclavicular, hip, knee, shoulder, and other joints [3-14]. Independent of co-occurrence with COVID-19, joint infections and cases of osteomyelitis (bone infections) can be serious consequences of associated sepsis, with a 6-16% mortality rate [15-17]. Septic arthritis can also lead to irreversible joint damage [15]. In addition, SEAs can also have mortality rates that approach 7%, as reported prior to the COVID-19 pandemic [18]. Although all strains of *Staphylococcus aureus *(*S. aureus*) are associated with increased mortality, there is an increased risk of patient death when experiencing co-infection with COVID-19 [19].

Significance of the case

We report the case of a healthy 62-year-old male patient who was hospitalized with mild COVID-19 with community-acquired methicillin-sensitive *S. aureus* (MSSA) co-infection. The patient developed MSSA bacteremia with concurrent bilateral shoulder infections and delayed spondylodiscitis with SEA. Only a few cases of *S. aureus* bacteremia and asymptomatic or mild COVID-19 co-infection are reported in the literature [3,20]. Similarly, only a few cases of shoulder infections without bacteremia in association with COVID-19 have been described. We located only two reported cases of unilateral shoulder infections (including septic arthritis in these cases) following COVID-19 (one adult and one infant) [21,22], and one reported case of bilateral shoulder infections in an adult [7]. Aside from cases of reactive arthritis caused by COVID-19, where non-infectious inflammation simultaneously occurs in various joints of the body [23,24], bacterial infection of multiple joints following COVID-19 is rare. In contrast, it is not rare to see multiple sites of deep-seated infection in cases of bacteremia or *S. aureus* syndromes independent of COVID-19 [25]. It is important that healthcare providers consider multiple locations of deep-seated infections as a potential complication of COVID-19 to avoid the serious consequences resulting from failure to detect and/or adequately treat these infections.

## Case presentation

A previously healthy, non-diabetic, non-smoker 62-year-old male (183 cm tall, 99 kg, BMI = 29.6) presented with a one-week history of mild left-side sciatica-like symptoms and a four-day history of moderate muscle pain with prominent involvement of his shoulders, hips, and thighs. He worked full-time as an orthopedic surgeon with fellowship training in shoulder and elbow surgery. His medical history included gastroesophageal reflux disease, for which he occasionally took omeprazole, and degenerative cervical disc disease, for which he occasionally took over-the-counter naproxen sodium 220 mg tablets. He had no history of arthritis or inflammatory conditions, no significant dysfunction of his shoulders or back/spine, no prior surgeries in these regions, and no chronic musculoskeletal pain disorders. He did not have any other medical problems. He had been vaccinated for COVID-19 on December 18, 2020, January 8, 2021 (Pfizer-BioNTech, mRNA), and November 3, 2021 (Moderna, mRNA).

He first began experiencing left-side sciatica-like symptoms in early September 2022 while assisting four workers from a moving company to lift and transport boxed items from his home to a new house. A chronology of subsequent symptoms, tests, and interventions appears in the chronology table (Table 1). A few years prior, he had similar left-side sciatica symptoms that were transient. Hence, he believed that the recent symptoms were exacerbated by his increased physical activity and reflected occult lumbar disc disease. However, no MRI scan had been done, and he had never sought medical attention for these symptoms. Two days later, he began experiencing moderate muscle pain, particularly in the truncal region and both shoulders, along with nocturnal fevers, chills, and sweats.

**Table 1:** Chronology of Symptoms, Tests, and Interventions

Main timeline	Date	Symptoms/tests/interventions
Before first hospitalization	September 2, 2022	Left-side sciatica-like (back/leg/foot) symptoms; increased physical activity believed to exacerbate symptoms of underlying lumbar disc disease
	September 4, 2022	Pain in both shoulders and truncal region; nocturnal fevers, chills, and sweats; no respiratory symptoms or typical COVID-19-related symptoms
	September 6, 2022	Bilateral thigh pain, persistent back pain, and increased bilateral shoulder pain; self-medicating with NSAIDs and steroid taper
	September 9, 2022	Gluteal intramuscular ketorolac injection; shoulder stiffness and pain begin preventing daily functions
First hospitalization (September 11-21, 2022)	September 11, 2022	Admitted to the hospital; difficulty walking; unable to raise arms above chest level; lumbar spine MRI - no evidence of infection; diagnosed with COVID-19 via PCR testing; treated with remdesivir; blood cultures reveal MSSA bacteremia
	September 12, 2022	MRI of brain - no evidence of an acute intracranial abnormality; MRI of right shoulder - moderate effusion of acromioclavicular joint and subacromial space; MRI of cervical spine - no evidence of infection; MRI left shoulder done Sept. 16
	September 13, 2022 (right shoulder surgery)	Aspirations: 1) MSSA from right subacromial space, and 2) Cutibacterium acnes from left acromioclavicular joint; aspiration glenohumeral joints - no growth; arthroscopic surgical debridement right shoulder; IV cefazolin administered
	September 14, 2022 (left shoulder surgery)	MRI of lumbar spine – no evidence of infection; arthroscopic surgical debridement left shoulder; trans-thoracic and trans-esophageal echocardiograms and myocardial PET/CT scan - normal cardiac function and no endocarditis
	September 15, 2022	Normal CT angiogram of abdomen and pelvis; CT scan for kidney stones is negative
	September 16, 2022	MRI of right shoulder - possible residual subacromial infection; MRI left shoulder - possible residual acromioclavicular joint infection
	September 17, 2022 (bilateral shoulder surgeries)	Open surgical debridements of both shoulders
	September 21, 2022	Discharged from the hospital; the plan is IV antibiotics for six weeks
Between hospitalizations	October 4, 2022	ESR increases, left-side sciatica symptoms improving
	October 5, 2022	Right-side sciatica symptoms appear
	October 11, 2022	ESR and CRP increase; polymyalgia rheumatica (PMR) considered
	October 21, 2022	Rheumatologist deems PMR unlikely
	October 25, 2022	Due to worsening leg and back pain, a repeat MRI of the lumbar spine was obtained; it shows L5-S1 discitis with osteomyelitis of the adjacent vertebral bodies with associated epidural abscess
Second hospitalization (October 26-30, 2022)	October 26, 2022 (spine surgery)	Surgical debridement of epidural material and entire L5-S1 intervertebral disc; tissue cultures - no growth (patient still on IV antibiotics); after surgery: two additional weeks of IV cefazolin, followed by four weeks oral linezolid
	October 30, 2022	Discharged from the hospital.
After second/final hospitalization	November 8, 2022	Normalization of CRP and WBC; patient progressively improved in all respects
	December 15, 2022	Patient returns to work duties
	November 26, 2024	Patient at 80% of his prior work capacity; experiencing persistent right foot and leg neuropathy, mild back pain, and continued mild bilateral shoulder pain

Five days later, he again assisted the moving crew and noted bilateral thigh pain, persistent back pain, and increased bilateral shoulder pain. During that time, he had been self-medicating for what he considered to be activity-related soreness/inflammation with (1) over-the-counter naproxen sodium 220 mg tablets twice a day, and (2) a six-day methylprednisone dose pack taper (4 mg tablets; six tablets taken on the first day, and one less tablet per day thereafter), which he self-prescribed and had taken for three days (15/21 tablets) by Day 8 of symptoms. On Day 7 of symptoms, he received a gluteal intramuscular Toradol® injection (ketorolac tromethamine; a nonsteroidal anti-inflammatory drug (NSAID)) for his musculoskeletal symptoms. At that time, his shoulder stiffness and pain were so severe that even simple tasks, like raising his arm to turn the key in his car’s ignition, became difficult.

Because of increasing bilateral shoulder pain, difficulty walking, inability to raise his arms above chest level (secondary to pain and stiffness), and generalized substantial musculoskeletal pain, the patient was admitted to the hospital nine days after symptom onset (September 11, 2022). It was initially suspected that he had rhabdomyolysis associated with his recent increase in physical activity; however, his myoglobin and creatinine kinase levels were normal. He was diagnosed with COVID-19 via polymerase chain reaction testing. Of note, the patient did not have any respiratory symptoms during the entire illness. According to NIH criteria (National Institutes of Health, USA), he had mildly symptomatic COVID-19, characterized by significant muscle pain and fatigue, without the respiratory symptoms that are typical of this illness (Table 2) [26].

**Table 2:** NIH Criteria for Assessing SARS-CoV-2 Infection Severity *The criteria for each category may overlap or vary across clinical guidelines and clinical trials, and a patient’s clinical status may change over time. SpO2 is a key parameter for defining the illness categories listed above [26]. NSAID: nonsteroidal anti-inflammatory drug; SpO2: oxygen saturation; NIH: National Institutes of Health

Severity	Criteria^*^
Asymptomatic or pre-symptomatic infection	Individuals who test positive for SARS-CoV-2 using a virologic test (i.e., a nucleic acid amplification test (NAAT) or an antigen test) but have no symptoms consistent with COVID-19.
Mild illness	Individuals who have any of the various signs and symptoms of COVID-19 (e.g., fever, cough, sore throat, malaise, headache, muscle pain, nausea, vomiting, diarrhea, loss of taste and smell) but do not have shortness of breath, dyspnea, or abnormal chest imaging.
Moderate illness	Individuals who show evidence of lower respiratory disease during clinical assessment or imaging and who have an oxygen saturation measured by pulse oximetry (SpO2 ) ≥94% on room air at sea level.
Severe illness	Individuals who have a SpO2 <94% on room air at sea level, a ratio of arterial partial pressure of oxygen to fraction of inspired oxygen (PaO2/FiO2) <300 mm Hg, a respiratory rate >30 breaths/min, or lung infiltrates >50%.
Critical illness	Individuals who have respiratory failure, septic shock, or multiple organ dysfunction.

**Figure 1:**
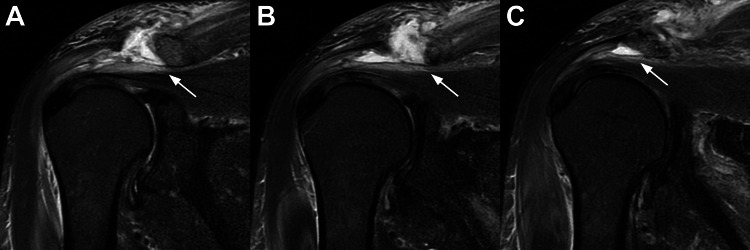
Coronal MR images of the patient’s right shoulder Coronal MR images of the patient’s right shoulder taken five days after the initial hospitalization (September 16, 2022). Each image is 4 mm apart, with A being the most anterior and C the most posterior slice. The white arrow indicates the subacromial infection.

Two sets of blood cultures revealed MSSA bacteremia. Non-contrast enhancing MR imaging of his cervical spine, thoracolumbar spine, brain, and shoulders was obtained to determine the source of the bacteremia. Based on obvious abnormal MRI findings shown in Figure 1, aspirations were done of the right subacromial space and left acromioclavicular joint. The results showed bilateral shoulder infection: (1) the right side with MSSA in the subacromial space, and (2) the left side with *Cutibacterium acnes* in the acromioclavicular joint. There was no evidence of crystals in the aspirates. Aspirations of his shoulder (glenohumeral) joints showed no growth from what appeared to be 1-2 cc of normal synovial fluid from each joint.

MR imaging (without contrast) of his lumbar spine done on the day of his admission and three days later (with IV contrast) did not show evidence of infection but did show mild-to-moderate right-sided disc bulges that did not correlate with his left-side sciatica-like symptoms. Six physicians (including two radiologists, two orthopedic surgeons, an internal medicine physician, and an orthopedic oncologic surgeon) examined his lumbar MR scans and did not detect evidence of infection. Brain and cervical spine MR scans also showed no signs of infection, and computed tomography (CT) angiograms of his abdomen and pelvis were normal.

The first surgical debridement was done four days later (September 13). The patient was started on IV cefazolin 2 g every eight hours as per standard of care, and underwent surgical debridement procedures for his shoulders. The patient remained bacteremic for five days, and blood cultures were negative on the seventh hospital day (September 17). COVID-19 treatment was with remdesivir [27]. During this hospitalization, he had additional imaging studies, including: (1) trans-thoracic and trans-esophageal echocardiograms, and (2) a myocardial positron emission tomography (PET)/CT scan. These studies showed normal cardiac function and no sign of endocarditis. The patient was discharged to his home on September 21, 2022, to complete a six-week course of cefazolin.

**Figure 2:**
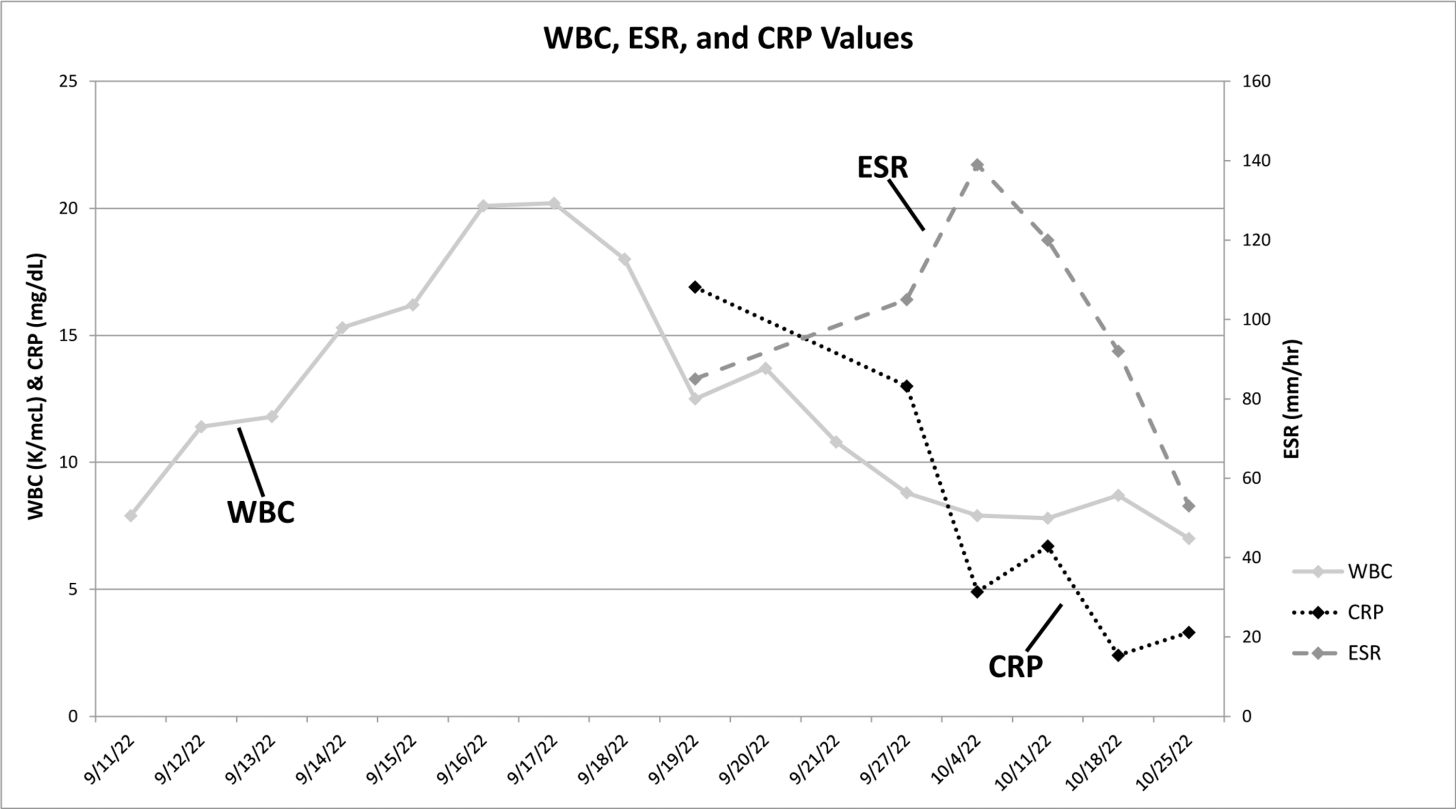
White blood cell count (WBC), erythrocyte sedimentation rate (ESR), and C-reactive protein (CRP) values White blood cell count (WBC), erythrocyte sedimentation rate (ESR), and C-reactive protein (CRP) values taken during the patient’s initial hospitalization until just prior to the spinal debridement surgery. Normal ranges for these values: WBC (3.6-10.6 K/mcL), ESR (0-20 mm/hr), CRP (<0.5 mg/dL). Note that: (1) all ESR values are elevated, (2) the second through tenth WBC values are elevated, and (3) all CRP values are elevated; this normalized by 11/8/2022 (not shown).

Figure 2 is a plot of his infection markers from September 11 to October 25, 2022. This figure shows persistent elevations in erythrocyte sedimentation rate (ESR) and C-reactive protein (CRP) values during his illness. From October 4 to 11, 2022, his ESR and CRP continued to increase, though the left-side sciatica symptoms were improving. On October 5, he began experiencing right-side sciatica-like symptoms, including high-level pain radiating to his right foot and numbness in the area. One week later, both ESR and CRP levels increased. These new symptoms and blood test results were discussed with the patient’s brother, who is also a physician, who raised the concern for possible polymyalgia rheumatica (PMR). The following week, the patient saw a physical medicine and rehabilitation physician who agreed that PMR was a reasonable possibility for the increased ESR values, sciatica, truncal-like symptoms, and shoulder and thigh pain [28].

**Figure 3:**
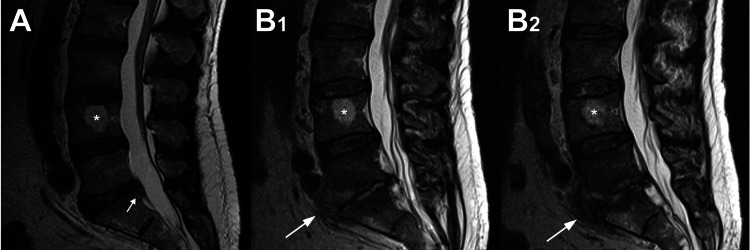
Sagittal MR images of the patient’s lumbar spine Sagittal MR images of the patient’s lumbar spine taken on (A) the day of hospital admission on September 11, 2022, and (B1-2) immediately prior to the epidural abscess debridement surgery on October 25, 2022. B2 is 6mm medial to B1. The large white arrows indicate the L5-S1 disc space where extensive discitis and the epidural abscess can be seen. The small arrow in A indicates a posterior disc bulge that may have contributed to the patient’s initial sciatic pain. The asterisk indicates a hemangioma found in the L4 vertebral body; several more were noted more proximally in the spine.

On October 21, 2022, a rheumatologist examined the patient and all aspects of his case and considered PMR to be unlikely. The patient was negative for rheumatoid factor and did not have elevated antinuclear antibodies. Because of worsening leg and back pain, repeat non-contrast MR imaging of the patient’s lumbar spine was done on October 25, 2022. These images showed findings consistent with L5-S1 discitis with osteomyelitis of the adjacent vertebral bodies and an associated epidural abscess, which is shown clearly in Figure 3. The patient was then re-admitted to the hospital and had surgical debridement of phlegmonous epidural material and of the entire L5-S1 intervertebral disc (with anticipation of eventual “auto-fusion” of these two vertebrae) [29]. Tissue cultures from the spinal disc and epidural tissue showed no growth; notably, the patient was still receiving IV cefazolin at that time. After the spinal debridement surgery, treatment included two additional weeks of IV cefazolin followed by four weeks of oral linezolid 600 mg twice a day. Linezolid was used to provide a highly bioavailable oral agent, allowing the cessation of IV therapy, and preventing complications from prolonged cefazolin and peripherally inserted central catheter (PICC) line use [30,31]. Normalization of his CRP and white blood cell (WBC) values occurred seven days after the spine surgery and the patient progressively improved in all respects.

**Figure 4:**
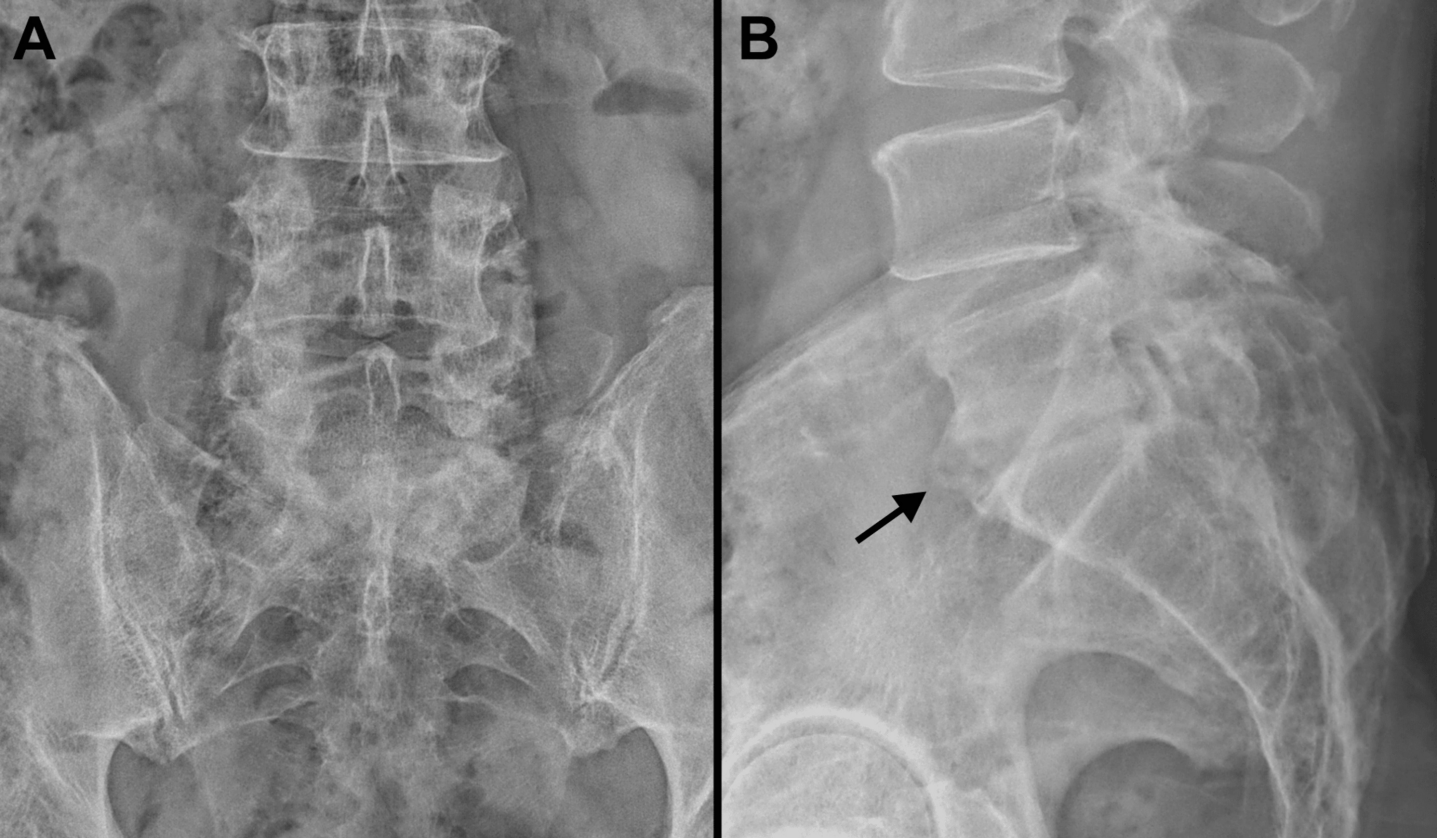
Final radiographs of the patient’s lumbar spine Anterior-posterior (A) and lateral (B) radiographs of the patient’s lumbar spine taken 20 months after his spine debridement surgery. Note that there is evidence of fusion at the L5-S1 intervertebral space (indicated by the black arrow in B).

He returned to his work duties as an orthopedic surgeon 95 days after his initial (September 11) hospitalization. At the time of completing this report, he was 25 months from his October 2022 spinal debridement surgery. He was at approximately 80% of his prior work capacity, which reflected relatively reduced physical endurance, persistent right foot and leg neuropathy (numbness, but full strength), and mild back pain. He also had continued mild pain in both shoulders, especially when reaching upward. Radiographs of his lumbar spine at 20 months after his spine debridement surgery show evidence of auto-fusion of the L5-S1 disc space, which is a favorable finding (Figure 4) [29].

## Discussion

This is the only case that we could locate in which a patient with mild COVID-19 had concurrent bacterial infections of both shoulder regions and spondylodiscitis/SEA. His mild COVID-19 presented with musculoskeletal symptoms. Although respiratory presentations of COVID-19 generally predominate, muscle and joint pain (myalgia/arthralgia) in COVID-19 are common presenting symptoms, with studies reporting an incidence ranging from 15 to 89% in cases of varying severity [32-34].

We examined studies and reports describing patients who had COVID-19 and a concurrent bacterial infection in the bloodstream or musculoskeletal system [3-10,14,20,21,35-72]. In these publications, we tallied the number of patients who had bacteremia (various species) and/or septic arthritis and/or a spinal infection in association with COVID-19 of any severity, including asymptomatic cases. Notably, in some cases, COVID-19 occurred several weeks before these clinical findings occurred. Excluded cases included those where bacteremia may have been caused by a clear nosocomial source such as an infected indwelling catheter. The goal of our literature overview was to determine the percentage of patients who met these criteria and were asymptomatic or, like our patient, had mild illness. We found 90 patients who met these broad criteria, and 11 (12.2%) of these patients were asymptomatic or had mild symptoms. Since the estimated incidence is likely higher than the actual rate due to reliance solely on reported cases, we expanded our search criteria to include asymptomatic or mildly symptomatic patients with bacterial infections from confirmed or presumed nosocomial sources. We located 11 additional patients, which dropped the estimated incidence to 11.9% (12/101) [38,42,43,61,65,67,68,73]. One patient with mild COVID-19 had an infected venous catheter [42]. Our patient is therefore in this smaller subset of patients where this association has been described.

In the studies and reports that we reviewed, 10 patients were identified with monomicrobial MSSA bacteremia, septic arthritis, SEA, or spondylodiscitis in association with COVID-19 of any severity [3,5,8,10,20,36,44,51,66]. An additional four patients with COVID-19 of any severity were reported to have monomicrobial *S. aureus* bacteremia, septic arthritis, SEA, or spondylodiscitis but neither MSSA nor MRSA was explicitly stated [6,39,40,47]. Of these 14 patients with this monomicrobial infection, three (21%) were associated with mild or asymptomatic COVID-19 [3,20]. For these three patients, we assessed whether they had medical problems or conditions or were chronically taking medications that are known to increase the risk of infection (e.g., advanced age (>80 years old), type 1 diabetes, alcoholism, significant smoking history, immunocompromising conditions such as rheumatoid arthritis or other autoimmune conditions, corticosteroid use, hepatitis, obesity, poor nutritional status, cancer, and etc.). Unlike our patients, all three patients had at least one risk factor, which included diabetes and iron deficiency anemia. Therefore, our patient’s case can be considered unique in this perspective. Interestingly, of the 11 patients who had moderate-to-critical COVID-19 severity and had MSSA bacteremia, non-specified *S. aureus* bacteremia (where MRSA was not explicitly stated), SEA, septic arthritis, or spondylodiscitis, five did not have any of the above mentioned medical problems/conditions [5,6,39,47,66].

Prior cases of bilateral shoulder infections in adults with COVID-19

The only case of an adult with COVID-19-related bilateral shoulder infections was reported by Neves et al. [7]. Their patient was a previously healthy 28-year-old male who, unlike our patient, had a severe case of COVID-19 that required mechanical ventilation. In the setting of severe disease and hospitalization, he developed bacterial pneumonia (*E. coli* and MSSA) and was treated with oral antibiotics. Seven days after discharge from the hospital, he developed thoracic and bilateral shoulder pain. MR scanning of both shoulders was suggestive of septic arthritis with contiguous fluid collections in the scapular fossae. Treatment was non-surgical and included aspirations and IV antibiotics [7].

Delayed diagnosis of spondylodiscitis and the use of PET/CT scanning in undetected acute cases

The most concerning aspect of our patient’s case was the delayed diagnosis of spondylodiscitis/SEA. By the time he had surgical debridement of his spinal infection, the patient had been on IV cefazolin for 5.5 weeks, the SEA was phlegmonous, and the L5-S1 disc was significantly compromised. At 24 months after spinal debridement surgery, the patient continues to experience persistent low-grade pain in his lower back and right lower leg, and foot numbness. It is possible that our patient developed lumbar spondylodiscitis very early in its course when MR scans of that region were taken on the first and fourth days of his initial hospitalization [74]. Bacteria had likely hematogenously seeded to the vertebral endplates, allowing the infection to spread to adjacent vertebral bodies (i.e., osteomyelitis) and the intervertebral disc, subsequently forming a SEA [75]. The infection may have evaded eradication with IV cefazolin because of relatively poor blood flow to the lumbosacral intervertebral discs [76].

Spinal epidural abscess is a rare diagnosis in the general population, with an incidence of 2-8 cases per 10,000 hospital admissions, as reported in a review article written without reference to the COVID-19 pandemic [77]. There is evidence that the incidence of SEA increased dramatically during the COVID-19 pandemic. For example, Talamonti et al. [3] noted that their institution had seen only seven cases of primary spondylodiscitis/SEA in the 10 years prior to the COVID-19 outbreak, but had seen six patients with this diagnosis within a three-month period (March 2020-May 2020) after the pandemic’s inception. The increased incidence of SEA during the COVID-19 pandemic raised concern because spondylodiscitis/SEA can cause spinal nerve compression and vascular damage that can potentially lead to paraplegia, quadriplegia, or even death [18,54,77].

Diagnosis of spondylodiscitis/SEA in our patient was very delayed, as this diagnosis was made 45 days after his initial hospital admission. The diagnosis of SEA is known to be elusive. Traditionally, SEAs are diagnosed based on clinical symptoms, laboratory testing, imaging studies, and invasive diagnostic tests. The “classic triad” for diagnosis includes spinal pain, fever, and neurologic deficit, which are nonspecific and can lead to misdiagnosis. Additionally, the clinical presentation is varied, as all three symptoms are present in only a minority of SEA patients [18,78]. This is further complicated by COVID-19, as patients may already be demonstrating body aches, muscle pains, and fever. The gold-standard diagnostic study for detecting SEA is gadolinium-enhanced MR imaging, which shows a greater than 90% sensitivity and specificity for detecting SEA [79]. However, such studies done during our patient’s acute presentation in the hospital did not reveal infection. It is currently recommended that patients who have clinical manifestations suggestive of spondylodiscitis/osteomyelitis have a repeat MRI two to four weeks after the onset of symptoms [80]. Studies have shown that compared to MRI, 18F-fluorodeoxyglucose (FDG) positron emission tomography and computed tomography (PET/CT) provide superior diagnostic value for detecting early spondylodiscitis [81]. This would have been beneficial for our patient, as earlier detection would have resulted in earlier surgical intervention.

COVID-induced immunosuppression

Previously healthy patients can become prone to secondary infections because of the immunosuppression and related impairments that can occur in COVID-19 [82,83]. For example, COVID-19 is known to be associated with dysregulation of the immune system by decreasing lymphocytes, with T-cell lymphocytes being particularly affected [84,85]. Functional T-cell exhaustion results from constant viral stimulation [85], thus impairing immune responses that in turn predispose some individuals to secondary infection and/or bacteremia [20]. While our patient’s CD4/CD8 T-cell counts were not measured, he had low lymphocyte counts ranging from 4-13% (normal = 25-33%) during and several weeks after his hospitalization. However, it should be noted that most cases of significant immunosuppression caused by COVID-19 have been reported in more severe cases than seen in our patients.

Classic risk factors for SEA include diabetes, alcohol use disorder, immunosuppression, intravenous drug use, trauma, and a primary locus of infection [77,86-88]. Because our patient had COVID-19 with concurrent MSSA bacteremia, it seems most parsimonious to conclude that his spondylodiscitis/SEA and bilateral shoulder infections were the result of hematogenous bacterial seeding. In a retrospective study prior to the COVID-19 pandemic, Vakili and Crum-Cianflone [18] found that 26 of their 101 cases of SEA were caused by bacteremia. This study and that of Talamonti and co-workers [3] indicate a common etiology that lends credence to our conclusion that bacteremia caused our patient’s SEA. While our patient fit the demographic characteristics for patients most likely to experience SEA, he only had one classic risk factor, which was possible COVID-19-induced immunosuppression.

COVID-19 can worsen *S. aureus* co-infections

During our patient’s hospitalization, it was speculated that he had a highly virulent strain of *S. aureus*. However, there is evidence that strains with lower virulence can be rendered significantly more virulent in the setting of SARS-CoV-2 co-infection. For example, Lubkin and co-workers [19] collected *S. aureus* strains from co-infected patients with SARS-CoV-2 to study the disease outcome caused by the interaction of these two pathogens. They also employed a mouse model with this co-infection. They found that in both patients and mice, co-infection with an *S. aureus* strain lacking significant toxicity resulted in more severe disease during the early phase of infection, compared with infection with either pathogen alone. Thus, their rigorous study shows that SARS-CoV-2 infection can directly increase the severity of *S. aureus* infection.

Unclear chronology: bacteremia prior to the onset of COVID-19 vs. after COVID-19 via induced endotheliitis/immunosuppression

If our patient only had *S. aureus* bacteremia, then the multiple locations of deep-seated infections would not be surprising. It is unclear whether bacteremia or COVID-19 was first present in our patient. It is possible that the bacterial infections developed independently of COVID-19 and, perhaps, reflected a relatively virulent strain of* S. aureus*. There are multiple theories that provide possible explanations of how COVID-19 may have enabled the bacterial infection, which is provided below.

Approximately 25-50% of SEAs are believed to come from hematogenous spread into the epidural space, with the majority originating from more distant skin or soft tissue injections [89,90]. A possible source of the bacteremia that precipitated our patient’s spondylodiscitis/SEA was the gluteal intramuscular ketorolac injection that he received two days before he was initially hospitalized (notably the viral and *S. aureus* inoculums were also separated by two days in the mouse model of Lubkin et al. [19]). The rarity of an intramuscular injection as a cause of SEA is evident by the fact that there is only one report of a patient who underwent multiple intramuscular non-steroidal anti-inflammatory drug injections and eventually developed an SEA [91]. That case was thought to be secondary to hematogenous spread from a gluteal abscess at the site of the intramuscular injection. Because our patient did not have an infection at the site of his ketorolac injection, it seems unlikely that the injection caused his bacteremia. However, in the setting of having symptomatic COVID-19, inoculation with *S. aureus* from that injection is a plausible explanation in our patient’s case because it was the only known episode where a violation of his epithelial tissues occurred prior to his hospitalization.

Another possible explanation, unrelated to the ketorolac injection, is that bacteremia developed due to the endothelial damage that is known to occur with COVID-19 infection. This is known as COVID-19-induced “endotheliitis,” which is typically considered to be most relevant in patients with severe/critical COVID-19 [92-94]. It is speculated that this damage plays an important role in the potential development of secondary bacterial infections in patients with COVID-19 [3,9,10,46,51,52]. Gut microbiome dysbiosis in antibiotic-treated COVID-19 patients is also known to be associated with microbial translocation and bacteremia [95].

The idea that endotheliitis might explain the bacteremia seen in our patient is suggested by the hypothesized causality of the case reported by Göre et al. [52]. They described the case of a previously healthy 56-year-old male who developed IgA nephropathy and a SEA with spondylodiscitis in association with COVID-19. The authors point out that the spike protein of the virus uses the ACE2 receptor to gain entrance into cells. These receptors are widely expressed in the airways, lung parenchyma, vascular endothelium, kidneys, small intestine, and central nervous system. Since *S. aureus* grew in their patient’s blood and urine cultures, they felt it was likely that the virus centralized in the vascular endothelium and kidneys due to their high expression of ACE2 receptors. A bacterial infection resulted shortly after in the same areas. Perhaps our patient’s loci of infection reflected the increased propensity of bacterial seeding into regions with existing inflammation [96], which resulted from his increased physical activities prior to when he contracted COVID-19.

As in the patient described by Göre and co-workers [52], endotheliitis is considered to be most significant in patients with moderate-to-severe COVID-19 [94,97]. However, it has been speculated that clinically relevant endotheliitis can still be present, though less prominent, in some milder cases of COVID-19 [3,98,99]. It has been suggested that endotheliitis might be less pronounced in some cases because of early antiviral and/or corticosteroid treatments [100]; this might be applicable in our patient’s case. Notably, in their report of six patients with COVID-19 and SEA, Talamonti et al. [3] hypothesized that COVID-19-related endotheliitis led to bacterial colonization elsewhere in the body in all of their patients (including their two patients with asymptomatic COVID-19).

A case series by Mohamed Ramlee et al. [58] supports the hypothesis that SEA can be a delayed complication of COVID-19. In that report, the authors describe three patients (ages 60, 69, and 24 years old) who were diagnosed with SEA 10, 12, and 12 weeks following COVID-19, respectively. They speculated that COVID-19 enabled secondary bacterial infections in two of the patients and the reactivation of a latent tuberculosis infection in the third patient. Furthermore, suspicion for a similar endotheliitis-related etiology associated with COVID-19 was also raised in the case of a 65-year-old male who developed spondylodiscitis/osteomyelitis and paravertebral abscesses in the setting of MSSA bacteremia that occurred nearly one month after a severe case of COVID-19 [10].

Mild COVID-19 and the drug-induced immunosuppression theory

Immunomodulatory therapies (IMT) (e.g., corticosteroids, IL‑6 inhibitors, GM‑CSF inhibitors, methotrexate, calcineurin inhibitors, and antitumor necrosis factor agents) have been used to treat the hyperinflammatory response seen in severe or critical COVID-19 [101]. This can lead to secondary infections in patients with and without COVID-19 [102-104]. Our patient had received a three-day course of methylprednisone (an oral corticosteroid) prior to his September 2022 hospital admission which may have contributed to an increased likelihood of developing a severe bacterial co-infection.

## Conclusions

The patient described in this report appears to have had COVID-19 mainly involved the musculoskeletal system and was associated with MSSA bacteremia. The cause of his MSSA bacteremia was not determined, but the intramuscular injection given two days prior to his hospital admission could be a possible source. The alternative explanation, that invasion of this bacterial species was enabled by viral-induced endotheliitis, seems less likely given the fact that he had minor COVID-19 symptoms. Some degree of immunocompromise from COVID-19 and his concurrent use of methylprednisone may have increased the propensity for seeding bacteria into the three sites where he had soreness/inflammation from recently increased physical activities.

Spondylodiscitis/SEA and infections of joints and joint regions are serious diagnoses, posing major risks for morbidity and mortality. Consequently, it is vital that healthcare providers have a low threshold for evaluation of co-infection when a COVID-19-positive patient presents with high levels of localized musculoskeletal pain and bacteremia. We also emphasize the importance of early detection in improving patient outcomes. This is especially true in cases where spine-related symptoms might reflect elusive diagnoses such as spondylodiscitis and SEA.
